# High-Intensity Physical Activity During Late Adolescence Predicts Young Adult CT-Based Finite Element Bone Strength in Emerging Adulthood: Iowa Bone Development Study

**DOI:** 10.3390/children12091204

**Published:** 2025-09-09

**Authors:** Soyang Kwon, Kathleen F. Janz, Indranil Guha, Alex V. Rowlands, Oscar Rysavy, Punam K. Saha, Chandler Pendleton, Euisung D. Shin, Steven M. Levy

**Affiliations:** 1 Buehler Center for Health Policy and Economics, Department of Emergency Medicine, Feinberg School of Medicine, Northwestern University, Chicago, IL 60611, USA; 2 Department of Health and Human Physiology, University of Iowa, Iowa City, IA 52242, USA; kathleen-janz@uiowa.edu; 3 Department of Electrical and Computer Engineering, University of Iowa, Iowa City, IA 52242, USA; indranil-guha@uiowa.edu (I.G.); punam-saha@uiowa.edu (P.K.S.); 4 Diabetes Research Centre, College of Life Sciences, University of Leicester, Leicester LE1 7RH, UK; alex.rowlands@leicester.ac.uk; 5 Leicester Diabetes Centre, Leicester General Hospital, University Hospitals of Leicester NHS Trust, Leicester LE5 4PW, UK; 6 Alliance for Research in Exercise, Nutrition and Activity (ARENA), UniSA Allied Health and Human Perfomance, University of South Australia, Adelaide 5001, Australia; 7 Department of Biostatistics, University of Iowa, Iowa City, IA 52242, USA; oscar-rysavy@uiowa.edu; 8 Emergency and Family Medicine Residency Program, Christina Care Health Systems, Newark, DE 19716, USAdavideuisung.shin@christianacare.org (E.D.S.); 9 Department of Preventive and Community Dentistry, University of Iowa, Iowa City, IA 52242, USA; steven-levy@uiowa.edu

**Keywords:** cohort study, physical activity intensity, physical activity volume, adolescent bone health, young adult bone integrity, finite element analysis (FEA), computed tomography (CT), musculoskeletal fitness

## Abstract

**Highlights:**

**What are the main findings?**

- Using CT-based finite element analysis of the tibia, this study found that higher intensity physical activity during late adolescence and emerging adulthood is associated with stronger bone structure.

**What is the implication of the main finding?**

- Advanced imaging and biomechanical modeling improve bone health estimation.
- High-intensity physical activity during late adolescence and emerging adulthood improves bone strength.

**Abstract:**

Objective: This study investigated associations between physical activity (PA) during late adolescence and emerging adulthood and bone strength in emerging adulthood by utilizing advanced finite element analysis of computed tomography (CT/FEA) technology beyond the traditional dual-energy X-ray absorptiometry (DXA) method. Methods: This study included 266 participants (152 females) from the Iowa Bone Development Study. PA volume (average acceleration) and intensity (intensity gradient) metrics were calculated from ActiGraph accelerometer data collected at ages 17, 19, 21, and 23 years. Compressive modulus and compressive stiffness of the tibia were estimated at age 23 via CT/FEA of the tibia. Sex-specific linear regression models were used to evaluate associations between PA metrics and bone outcomes, adjusting for age, height, weight, musculoskeletal fitness, and calcium intake. Results: Intensity gradient averaged over 17–23 years of age was positively associated with compressive stiffness at age 23 years in both females and males (*p* < 0.01). Intensity gradient was positively associated with compressive modulus in females (*p* < 0.01), but not in males. No significant associations were found between average acceleration and either compressive stiffness or modulus in either sex (*p* > 0.05). Conclusions: Using a state-of-the-art CT/FEA method, this study suggests that high-intensity PA during late adolescence and emerging adulthood improves bone strength.

## 1. Introduction

Early adulthood marks the culmination of bone mass accrual, with peak bone mass typically attained during this period [1,2]. Low peak bone mass increases long-term risk for skeletal fragility and bone fractures [3,4,5,6,7,8,9,10,11,12]. A one standard deviation increase in bone mineral density is associated with approximately a 50% reduction in fracture risk among young adults aged 20–29 years [13]. However, fracture risk depends on more than just densitometry-measured bone mass, since the resistance of a specific bone to deformation is determined by a combination of material and structural properties [14,15]. Clinically, bone health has often been evaluated using imaging modalities such as dual-energy X-ray absorptiometry (DXA) and peripheral quantitative computed tomography (pQCT), which provide measures of bone mineral content, microarchitecture, and volumetric density [16]. These imaging-derived metrics serve as useful proxies for the combined effects of bone material and structure during deformation. However, it is stress during deformation, not bone mass or quality alone, that most closely predicts bone strength and fracture risk [17]. An advanced computer technique for structural stress analysis using 3-dimensional CT scan data, finite element analysis (FEA), enables estimation of internal stress and strain distributions, offering a more precise measurement of bone’s mechanical integrity and fracture risk [17]. Unlike DXA and pQCT which provide indirect estimates of bone strength based on density and geometry, CT-based FEA (CT/FEA) offers a direct biomechanical assessment by simulating how bone responds to mechanical loading, thereby providing a more functionally relevant measure of fracture resistance [18]. CT/FEA has been shown to provide more reliable surrogates of bone strength, compared to traditional densitometric measures, such as bone mineral content, bone mineral density, and areal bone mineral density [19,20,21,22]. CT/FEA-derived bone strength calculations have been used to estimate fracture risk and monitor treatment efficacy in osteoporosis [17].

Prior reports, including those from the Iowa Bone Development Study (IBDS), have consistently shown positive associations between physical activity (PA) and DXA- and pQCT-derived bone parameters in childhood through early adulthood [23,24,25,26]. Using the IBDS’s final follow-up data at age 23 years, Rowlands et al. [24] reported that both PA intensity and volume from ages 17 to 23 were positively associated with DXA-derived total body bone mineral content and hip bone mineral density at age 23 among females and males. To our knowledge, no prior human studies have investigated CT/FEA-derived bone strength measures in relation to free-living PA in young populations [27]. This present study builds on the prior work [24] by utilizing compressive modulus and stiffness measures, derived from CT-based FEA of the tibia (CT/FEA), to more directly quantify bone strength. CT/FEA-derived compressive modulus is a material-level property reflecting the elastic response of the bone tissue itself to compressive loading [28]. CT/FEA-derived compressive stiffness is a structural-level property that captures the overall resistance of the entire bone segment to compression [29].

This present study aimed to confirm and extend previous IBDS findings by linking accelerometer-based PA intensity and volume measures with these advanced CT/FEA-based bone strength measures. We hypothesized that both PA intensity and volume would be independently associated with bone compressive modulus and stiffness, with sex-specific effects. This article is a revised and expanded version of a paper entitled “High-magnitude physical activity and musculoskeletal fitness in youth predict young adult bone strength: Iowa Bone Development Study”, which was presented at the 2024 North American Society for Pediatric Exercise Medicine Conference in Louisville, Kentucky, USA, in August 2024. This study has important implications for sports scientists aiming to optimize training regimens for bone health, as well as public health professionals designing youth PA guidelines.

## 2. Materials and Methods

### 2.1. Study Sample

Participants were drawn from the IBDS, a longitudinal cohort study that included a subset of the Iowa Fluoride birth cohort study. The Iowa Fluoride Study recruited 1882 full-term newborns recruited from 8 hospital postpartum wards in Iowa, USA, between 1992 and 1995. Of them, 890 were recruited for the IBDS at approximate age 5 years. Approximately 95% of IBDS participants were non-Hispanic white. Approximately 70% of mothers had a 4-year college degree. IBDS conducted study assessments every 2–3 years from ages 5 through 23. Over 18 years of the follow-up period, 562 of 890 participants (63.1%) were lost to follow up and 328 (36.9%) participated in a study visit at age 23. For this report, we included 266 participants (29.9%) who completed a CT scan at age 23 and had measurements available for all considered explanatory variables. Those who were included in this analysis had similar demographic distributions, specifically in race/ethnicity, sex, and maternal education attainment, as well as similar PA levels at age 5, compared to those who were excluded.

### 2.2. Physical Activity Exposure

Exposure variables were accelerometer-measured average acceleration (PA volume indicator) and intensity gradient (PA intensity indicator) during late adolescence through emerging adulthood. Average acceleration is the mean acceleration across the 24-h day (mg) and intensity gradient is the distribution of intensity across the 24-h day [30]. Average acceleration and intensity gradient metrics have been used as summary metrics of a 24-h activity profile [31,32]. They are useful to explore the independent effects of PA volume and intensity [33]. To assess PA, participants were asked to wear a three-axis ActiGraph GT3X+ accelerometer (Pensacola, FL, USA) on the hip for five days, including both weekend days, at ages 17, 19, 21, and 23 years. At ages 17, 19, and 21 years, participants were instructed to wear the monitor during a waking day. At age 23 years, a 24-h wear was optional. Accelerometer data were processed to derive average acceleration and intensity gradient metrics using previously established protocols from IBDS analyses [24]. Briefly, accelerometer files were processed using R-package GGIR (version 1.9-4) [34] to detect non-wear (defined as at least 60 consecutive minutes of acceleration below a threshold, allowing up to 2 min of short interruptions) [35] and to calculate the average magnitude of dynamic acceleration using the Euclidean Norm Minus One (ENMO) metric [34,35]. Non-wear was imputed using the average at similar time points on different days of the week (default setting in GGIR). We calculated an average of all available values from four assessments under the assumption of missing at random [24] to represent cumulative PA exposure across late adolescence and early adulthood.

### 2.3. Bone Strength Outcomes

Outcome variables were tibial CT/FEA-derived compressive modulus and compressive stiffness. CT/FEA is a computational modeling technique that integrates high-resolution CT imaging with biomechanical simulations to estimate bone mechanical behavior under specific loading conditions [17,36,37]. FEA simulates the physical phenomenon of a specific mechanical loading condition on a target object using a finite network of mesh elements [38]. This method enables a non-invasive, subject-specific assessment of bone strength by simulating the mechanical response of bone tissue to forces that mimic physiological loading [39]. Among CT/FEA-derived bone strength metrics, we used compressive modulus and stiffness that represent functional properties of bone quality and fracture resistance. Compressive modulus characterizes its ability to resist deformation per unit stress and provides insight into the microstructural quality of the bone. Therefore, compressive modulus is useful for assessing bone quality and bone fragility at a microstructural level [40]. Higher compressive modulus values indicate stiffer, more mineralized bone tissue. Compressive stiffness quantifies the total resistance of the bone segment to axial deformation under compressive forces [29]. As such, compressive stiffness is a functional indicator of whole-bone mechanical competence and is directly related to fracture resistance under physiological loading [17]. Higher compressive stiffness values reflect greater structural integrity and an enhanced ability of bone to resist mechanical deformation. These two metrics together offer a comprehensive biomechanical assessment of bone strength beyond traditional bone density measurements alone [17]: compressive modulus informs on the quality of the bone matrix at the tissue level, while compressive stiffness provides an integrated measure of bone’s structural and functional performance.

**Figure 1:**
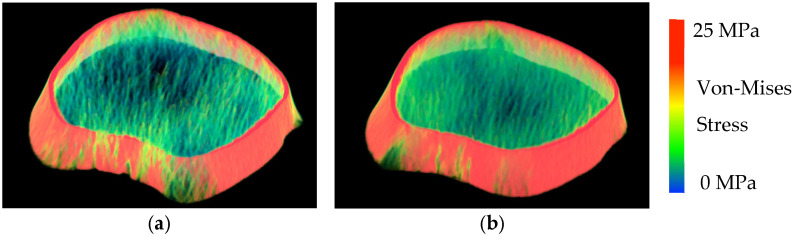
FEA-modeled images of the distal tibia from two female participants. (**a**). Stiffness: 451 kN/mm and Modulus: 2991 MPa. (**b**). Stiffness: 652 kN/mm and Modulus: 4409 MPa.

At age 23, participants underwent pQCT imaging of the distal tibia at 4–6% metaphyseal region using a Siemens SOMATOM Force scanner (120 kV, 100 mAs; Forchheim, Germany). The region of interest was chosen for its high trabecular content and relevance to lower limb loading. The imaging protocol employed a voxel resolution of 170 μm in-plane and a 200 μm slice thickness, optimized for precise representation of bone geometry and density. A calibration phantom was used to convert CT intensity numbers in Hounsfield units into volumetric bone mineral density values [38]. Voxel-based nonlinear continuum FEA was performed using the ANSYS software (ANSYS Mechanical 2019 R2, Ansys Inc., South Pointe, PA, USA; Figure 1). The inferior surface of the cylindrical volume of interest (VOI) was fully constrained, while a uniform displacement was applied in the z-axis to each node on the superior surface to simulate physiological axial compressive loading. A Poisson’s ratio of 0.3 was assumed. Trabecular bone modulus was calculated as the ratio of the average von Mises stress (MPa) across elements on the top surface to the applied displacement [38]. Compressive stiffness was derived by dividing total reaction force by applied displacement (kN/mm). Our prior experiments demonstrated that continuum FEA-derived metrics showed high reliability (intraclass correlation coefficient = 0.98) and strong correlations with microstructural reference values at in vivo imaging resolution (r ≥ 0.87) [38].

### 2.4. Other Measurements

To ascertain associations between PA volume and intensity metrics and bone structure metrics, we considered age, weight, height, and calcium intake (estimated from Diet History Questionnaire II) [41] measured at age 23. Additionally, we accounted for the effect of musculoskeletal fitness based on prior evidence linking musculoskeletal fitness to bone outcomes [42,43,44,45]. To assess musculoskeletal fitness, participants completed a vertical jump test and a handgrip strength test during assessment visits at ages 17, 19, and 23 years [46,47]. We used a Vertec (Questek Corp., Elgin, IL, USA) to measure vertical jump height in centimeters (cm). For the jump test, participants performed a squat jump by bending their knees and moving their arms behind them until their knuckles faced the floor, holding this squat position momentarily so as to eliminate countermovement momentum. They then jumped as high as possible while reaching up and hitting the Vertec vanes with the dominant arm. This test was repeated three times per person per wave and three jump height values (cm) were averaged. Then, jump power (Watts) was calculated by 60.7 × jump height (cm) + 45.3 × body mass (kg) − 2055 [48]. For handgrip strength, we used a Jamar hand dynamometer (Creative Health Products, Plymouth, MI, USA), following a standard protocol. Participants were seated with their elbow flexed at 90°, forearm in a neutral position and wrist between 0° and 30° dorsiflexion. Each hand was tested twice, and the maximum value in kilograms from each hand was recorded. The highest value from either hand was used as the final handgrip strength measure to reflect maximal voluntary contraction.

### 2.5. Statistical Analysis

All analysis was stratified by sex. Descriptive analyses, including frequency and distribution analyses, were conducted. Multivariable linear regression models were used to evaluate associations between PA volume and intensity and compressive modulus and stiffness outcomes. These models were adjusted for potential confounders measured at age 23: age, weight, height, and calcium intake (estimated from Diet History Questionnaire II) [41]. Musculoskeletal fitness was also included as a covariate, represented by average handgrip strength (kg) and vertical jump power (Watts) across ages 17, 19, and 23. Model assumptions and residuals were tested for model diagnosis. All analyses were performed in R version 4.4.

## 3. Results

Among the 266 participants (152 females) included in the analysis, the means of daily average acceleration across the four assessments were 15.0 mg (range of 4.1 to 25.9) for females and 15.8 mg (range of 7.1 to 28.1) for males (Table 1). The means of intensity gradient were −2.93 (range of −3.50 to −2.24) for females and −2.90 (range of −3.39 to −2.45) for males.

As presented in Table 2, in adjusted models, intensity gradient averaged over 17–23 years of age was positively associated with compressive modulus in females (*p* < 0.01), but not in males. Average acceleration was not associated with compressive modulus among either females or males.

As shown in Table 3, in adjusted models, intensity gradient averaged over 17–23 years of age was positively associated with compressive stiffness at age 23 years in both females and males (*p* < 0.01). No significant associations were found between average acceleration and compressive stiffness among either females or males.

**Figure 2:**
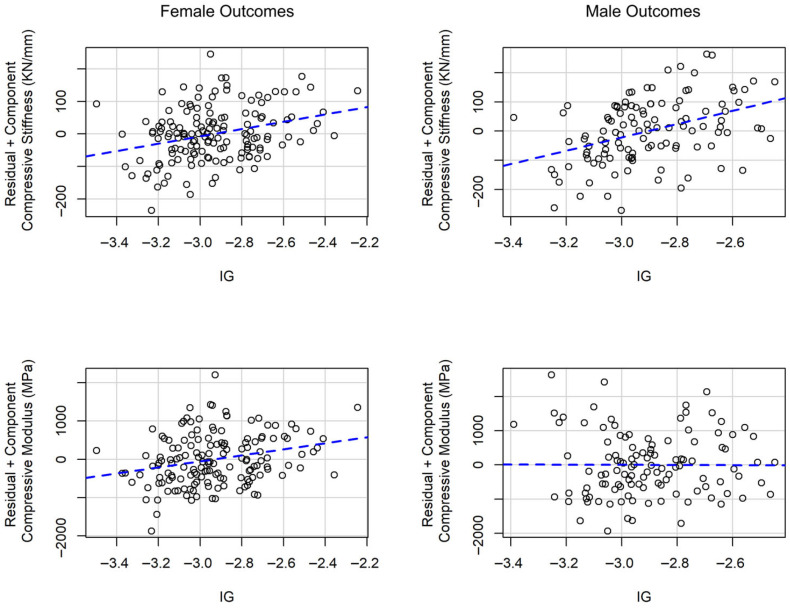
Partial residual plots for the relationship between intensity gradient (IG) and compressive modulus and stiffness in multivariable linear regression models.

Figure 2 presents partial residual plots derived from the multivariable linear regression models. These plots illustrate a linear positive relationship between intensity gradient and both compressive modulus and stiffness among females and between intensity gradient and compressive stiffness among males.

## 4. Discussion

**Summary of Findings.** Using advanced CT/FEA of the tibia, the study found that PA intensity during late adolescence through emerging adulthood was positively associated with compressive stiffness in emerging adulthood among both females and males. PA intensity was also positively associated with compressive modulus among females, but not among males. In contrast, PA volume showed no significant association with either bone strength outcomes.

**Consistency with prior IBDS findings and the broader literature.** These findings extend the substantial body of evidence from the IBDS, which has consistently demonstrated positive effects of youth PA, especially vigorous-intensity PA on bone outcomes using increasingly sophisticated measurement approaches [25,26]. More recently, Rowlands et al. [24] extended these findings to age 23 years by showing positive associations between both PA intensity and volume and DXA-derived hip bone mineral density and total body bone mineral content at age 23 in the IBDS sample. The study [24] also found that PA volume, but not intensity, was positively associated with DXA-derived hip geometry outcomes such as cross-sectional area and section modulus.

By incorporating a tibial CT/FEA method, the present study advances research by directly estimating bone strength under simulated mechanical loading. The study results showed the positive association between PA intensity and bone strength among young people. Specifically, this study highlights the importance of high-intensity PA during late adolescence through emerging adulthood, rather than total PA volume, on improving bone mechanical competence, as measured by compressive stiffness and modulus. Notably, in both females and males, PA intensity was positively associated with compressive stiffness, which is a clinically meaningful metric that directly reflects the bone’s ability to resist deformation and potential fracture [49]. The positive association between PA intensity and compressive modulus observed only in females, but not in males, may be partly explained by sex-specific differences in skeletal adaptation; specifically, testosterone-driven periosteal expansion in males could attenuate detectable effects on tissue-level properties such as modulus. This divergence from earlier DXA-based findings by Rowlands et al. [24] may reflect the greater sensitivity of FEA-derived bone strength metrics to mechanical loading characteristics, which are more directly influenced by activity intensity than total activity volume. High-intensity loading likely induces greater osteogenic stimuli by exceeding the mechanical thresholds necessary to trigger bone formation, particularly during the critical window of adolescence and emerging adulthood, thereby more effectively enhancing the structural and material bone properties captured by FEA. These findings support the role of high-intensity PA in stimulating bone mechanoadaptation [50,51] and align with recent evidence from systematic review [50] indicating that vigorous-intensity PA produces greater benefits for bone health than lower-intensity PA in youth. Taken together, our findings reinforce the concept that activity intensity and loading magnitude are key drivers of bone strength development.

**Implications.** These findings suggest that high-intensity PA, particularly movements that involve dynamic, high-magnitude loading, may be more effective in promoting bone strength and preventing future fractures rather than increasing PA volume. While musculoskeletal fitness (e.g., handgrip strength, jump power) remains important [42,43,44,45], our results emphasize the added value of bone-targeted loading patterns through high-intensity PA. Practical implications may include encouraging high-intensity physical activity that significantly elevate heart rate, such as school-based plyometrics, vigorous sports and dance, and running, during late adolescence. Future interventions aimed at optimizing skeletal health in youth and young adults should prioritize high-impact or high-intensity PA, rather than focusing on overall PA volume. These strategies may offer a more direct pathway to improve long-term bone strength.

**Limitations.** While the CT/FEA method is highly innovative, it is also sensitive to modeling assumptions, including boundary conditions, loading direction, and software-specific parameters, which may have influenced the absolute values of computed bone strength estimates. Residual confounding from unmeasured confounders, such as genetic predisposition, early-life PA exposure, or baseline bone health, may have biased the observed associations. Attrition over the two-decade study period could have introduced selection bias, limiting generalizability to the broader IBDS cohort. The predominantly non-Hispanic White sample from a Midwestern U.S. region also limits generalizability to racially, ethnically, or geographically diverse populations.

## 5. Conclusions

This study adds to the field by using CT/FEA to quantify functional bone strength metrics in emerging adults. Using high-resolution imaging and advanced biomechanical modeling, this study provides strong evidence that high-intensity PA during late adolescence and emerging adulthood is beneficial for bone strength. These findings support youth high-intensity PA as a critical component of evidence-based strategies to optimize bone strength.

## Figures and Tables

**Table 1:** Means and standard deviations (SD) of study variables by sex.

Variables	Females (*n* = 152)	Males (*n* = 114)
	Mean ± SD	Mean ± SD
Age (years)	23.5 ± 0.5	23.4 ± 0.6
Height (cm)	166.2 ± 7.0	180.0 ± 8.1
Weight (kg)	74.6 ± 20.3	91.3 ± 22.0
Calcium intake (mg/day)	818 ± 590	1218 ± 792
Handgrip strength ^a^ (kg)	28.3 ± 6.0	43.8 ± 10.0
Jump power ^a^ (Watts)	3557 ± 869	5104 ± 1023
Average acceleration ^b^ (mg)	14.97 ± 4.16	15.84 ± 3.92
Intensity gradient ^b^	−2.93 ± 0.22	−2.90 ± 0.20
Proportion worn ^b^ (%)	0.88 ± 0.09	0.87 ± 0.08
Compressive modulus (MPa)	3427 ± 695	4166 ± 978
Compressive stiffness (KN/mm)	506 ± 93	657 ± 137

Age, height, weight, calcium intake, compressive modulus, and compressive stiffness assessed at age 23 years are presented. ^a^ Three assessments at 17, 19, and 23 years were averaged. ^b^ Four assessments at 17, 19, 21, and 23 years were averaged. Intensity gradient is gradient of the regression line from log-log plot of intensity (x) and minutes accumulated (y).

**Table 2:** Multivariable linear regression models for compressive modulus (MPa) by sex.

	Female		Male	
Predictor	β	95% CI	β	95% CI
Age (years)	−18	−214, 179	128	−204, 459
Height (cm)	−18	−36, 1	−36 *	−64, −8
Weight (kg)	8	−3, 20	−10	−24, 4
Calcium intake (mg/day)	0.02	−0.2, 0.2	0.04	−0.2, 0.3
Handgrip strength (kg) ^a^	16	−6, 39	−12	−36, 12
Jump power (Watts) ^a^	0.01	−0.3, 0.3	0.3	−0.1, 1
Average acceleration (mg) ^b^	−9	−39, 21	27	−23, 77
Intensity gradient ^b^	785 **	222, 1348	−20	−1045, 1006

* *p* < 0.05; ** *p* < 0.01; β, coefficient; CI, confidence interval. Age, height, weight, and calcium intake assessed at age 23 years were used. ^a^ Three muscular fitness assessments at 17, 19, and 23 years were averaged. ^b^ Four accelerometer assessments at 17, 19, 21, and 23 years were averaged.

**Table 3:** Multivariable linear regression models for compressive stiffness (KN/mm) by sex.

	Female		Male	
Predictor	β	95% CI	β	95% CI
Age (years)	−8	−32, 17	14	−22, 50
Height (cm)	−2 *	−5, 0	−9 **	−12, −6
Weight (kg)	1	−0.1, 3	−0.4	−2, 1
Calcium intake (mg/day)	−0.001	−0.02, 0.02	0.03 *	0.004, 0.1
Handgrip strength (kg) ^a^	2	−0.4, 5	1	−1, 4
Jump power (Watts) ^a^	0.02	−0.02, 0.1	0.1 **	0.04, 0.1
Average acceleration (mg) ^b^	−1	−5, 3	1	−4, 7
Intensity gradient ^b^	113 **	44,182	228 **	117, 339

* *p* < 0.05; ** *p* < 0.01; β, coefficient; CI, confidence interval. Age, height, weight, and calcium intake assessed at age 23 years were used. ^a^ Three muscular fitness assessments at 17, 19, and 23 years were averaged. ^b^ Four accelerometer assessments at 17, 19, 21, and 23 years were averaged.

## Data Availability

The original contributions presented in this study are included in the article. Further inquiries can be directed to the corresponding author.

## Abbreviations

The following abbreviations are used in this manuscript:

CI	confidence interval
CT	computed tomography
CT/FEA	computed tomography-based finite element analysis
DXA	dual-energy X-ray absorptiometry
FEA	finite element analysis
IBDS	Iowa Bone Development Study
PA	physical activity
pQCT	peripheral quantitative computed tomography
